# A DNA vaccine based on hemagglutinin and conserved epitopes of influenza B virus provides cross-lineage protection in mice

**DOI:** 10.3389/fimmu.2025.1645744

**Published:** 2025-10-29

**Authors:** Xiangyu Zhu, Zhuo Ha, Siyang Liu, He Zhang, Wenxin Zhao, Xiangshu Qiu, Xinyu Cao, Wei Wang, Yubiao Xie, Ning Shi, Jicheng Han, Wei Zheng, Huijun Lu

**Affiliations:** ^1^ College of Veterinary Medicine, Jilin Agricultural University, Changchun, Jilin, China; ^2^ State Key Laboratory of Pathogen and Biosecurity, Key Laboratory of Jilin Province for Zoonosis Prevention and Control, Changchun Veterinary Research Institute, Chinese Academy of Agricultural Sciences, Changchun, Jilin, China; ^3^ Faculty of Chemistry, Northeast Normal University, Changchun, Jilin, China; ^4^ Wenzhou University, Wenzhou, Zhejiang, China; ^5^ Key Laboratory of Jilin Province for Traditional Chinese Medicine Prevention and Treatment of Infectious Diseases, College of Integrative Medicine, Changchun University of Chinese Medicine, Changchun, Jilin, China; ^6^ The 964th Hospital of the PLA Joint Logistics Support Force, Changchun, Jilin, China

**Keywords:** influenza B virus, DNA vaccine, electroporation, cross-protection, conserved epitopes

## Abstract

Conventional influenza vaccine can prevent infection and reduce the risk of post-infection complications. However, they lack the capacity to effectively respond to influenza virus mutations. This results in the vaccine becoming ineffective due to a reduced antigenic match. It is necessary to develop a new strategy for vaccine that will provide broad cross-reactive protection. A DNA vaccine based on the hemagglutinin (HA) gene and conserved antigenic epitopes of both the HA, M2e and NA genes to provide protection against influenza B was developed. BALB/c mice were immunized with electroporation to evaluate both humoral immune responses and T cell responses. Protection against influenza B virus challenge was evaluated in DNA vaccinated mice, followed by analysis of lung tissue to assess changes in cytokine levels and virus load. Additionally, various assays with DNA were conducted to assess their cellular uptake by DCs and their potential for immune activation. Vaccine *via* electroporation demonstrated the ability to enhance both humoral and cellular immune responses and resulted in the shaping of the immune response to the vaccine in a Th1 direction. Animals inoculated with vaccines *via* electroporation were completely protected against both homologous and heterologous viruses, as evidenced by the reduction of lung viral loads and lung inflammation, induction of broadly cross-protective humoral immunity, and IL-2 CD4^+^ T-cell responses. The most significant finding was that the DNA vaccine provided complete protection for mice against two distinct lineages of the lethal influenza B virus. These findings suggested that DNA vaccine delivered using *in vivo* electroporation effectively elicits a protective immune response and provides additional cross-protection.

## 1 Introduction

Seasonal influenza, caused by influenza viruses, is a contagious viral disease that causes annual epidemics and occasionally leads to global pandemics. The scope of the illness places a heavy burden on public health systems and resulting in the hospitalization of more than five million adults each year (1).Seasonal influenza is caused by two distinct types of viruses: influenza A virus (IAV) and influenza B virus (IBV). These viruses alternately or co-infect humans and are similar in terms of their clinical severity (2). Since it was first described in the 1940s, IBV has gradually differentiated into two distinct lineages - B/Victoria/2/87 (Victoria lineage) and B/Yamagata/16/88 (Yamagata lineage) (3). Until March 2020, the two lineages alternated epidemics caused by IBV or IAV epidemics, significantly increasing the global burden of influenza disease. Following the onset of the COVID-19 pandemic, the B/Victoria lineage became the predominant endemic strain, while the B/Yamagata lineage was rarely detected. The underlying reasons are unclear (4, 5).

There are two types of licensed influenza drugs: neuraminidase inhibitors (NAIs) and amantadine. However, both drug types have poor inhibitory activity against IBV, in part due to drug-resistant mutations that develop and persist in IBV (6, 7). Monoclonal antibody CR9114 is currently the only protective antibody that can cross-react with IAV. This cross-reactivity broadly prevents IAV and IBV disease (8). While existing pharmaceuticals can effectively inhibit viral replication, patients with severe influenza cannot be adequately treated with existing drugs. Prevention and control of influenza must begin with the blocking of the source of viral infection. Currently, immunization vaccines remain the primary strategy for interrupting viral infection. Vaccines are significantly less expensive than drug treatment of ongoing infections. The most widely used influenza vaccines are the trivalent (H1N1, H3N2, B/Victoria) and quadrivalent (H1N1, H3N2, B/Victoria, B/Yamagata) seasonal influenza vaccines produced using World Health Organization recommended vaccine strains. These vaccines effectively protect healthy adults against well-matched strains. However, due to the high antigenic variability of influenza surface antigens, mismatches are common and vaccine effectiveness is compromised (9–12). The development of new strategies for universal influenza vaccines is imperative to combat the emergence of multiple variants.

A variety of strategies have been explored. Several have considerable promise. These include the use of epitopes that are conserved across different influenza virus strains as vaccine immunogens (13–15). This approach is supported by a substantial body of research, with numerous studies highlighting the potential of conserved structures derived from HA, matrix-2 (M2), and nucleoprotein (NP) in the development of a universal influenza vaccine (16). Conserved antigens, such as the ectodomain of the influenza M2 protein, M2e, or the HA stalk domains, typically evoke weak immune responses. To enhance their potency, these antigens require the addition of adjuvants (16, 17). IBV vaccine studies have been mainly limited to evaluating the combination of conserved epitopes of HA and neuraminidase (NA) (18–20). Fewer studies have addressed M2 and NP, which are two highly conserved IBV proteins (21). Compared to virus-like particles and protein-based or mRNA vaccines, DNA vaccines have a relatively simple production process and can be scaled-up for mass production (22, 23). In addition, DNA vaccines are very stable (24), which is a favorable quality compared to other vaccine platforms in terms of storage and transportation conditions (25, 26). Accordingly, it is plausible that a DNA vaccine for influenza may prove an efficacious method of preventing the disease.

In this study, we used a DNA vaccine model to investigate the *in vivo* immunogenicity of IBV full-length HA in tandem with HA, M2e, and NA antigenic epitopes as antigens. Intramuscular injection of plasmid DNA was followed by *in vivo* electroporation delivery. The cross-protection of the vaccine against a different lineage of IBV was evaluated. The findings provided a theoretical basis for the design of an influenza vaccine strategy based on full-length HA groups and antigenic epitopes. The current findings could facilitate the development of multivalent vaccines with enhanced immunogenicity and effective cross-immunity.

## 2 Materials and methods

### 2.1 Mice, cells and viruses

Female BALB/c mice (6-8-week-old) were purchased from Beijing HFK Bioscience Co., Ltd. The Human embryonic kidney (HEK 293) cells were maintained in our laboratory. HEK 293 cells were cultivated in Dulbecco's Modified Eagle's medium (Gibco, USA) augmented with 10% fetal bovine serum (FBS; Gibco, USA) and a solution containing penicillin (100 U/mL) and -streptomycin (100 µg/mL). To harvest bone marrow, BALB/c mice were euthanized by cervical dislocation after being anesthetized with an intraperitoneal injection of tribromoethanol (2.01%, 20 μL/g body weight). Bone marrow was obtained from the femur and tibia of mice and washed with RPMI-1640 medium (Hyclone, USA). After filtration through a 70 μm filter, the cells were cultured in RPMI-1640 medium containing 100 U/mL penicillin and 100 μg/mL streptomycin. The culture medium was supplemented with 20 ng/mL recombinant murine granulocyte-macrophage colony-stimulating factor, 10 ng/mL recombinant IL-4, and 10% inactivated FBS at 37°C in an atmosphere of 5% CO_2_. The medium was replenished half-way through the incubation period. This process yielded mouse bone marrow-derived DCs dendritic cells. B/Chicken embryos/2022 (Victoria) (GenBank: OR775570) and B/Massachusetts/2/2012 (Yamagata) (GenBank: MT056027) were obtained from our laboratory collection. All experiments involving infectious IBV were performed in a level-2 facility.

### 2.2 Construction of plasmid DNA

The HA gene of IBV Victoria lineage B/Austria/1359417/2021 (GISAID no. EPI1926631) was codon-optimized for human expression. The optimization process involved changes to the GC content, codon usage, distribution, and incorporation of restriction enzyme recognition sites. Approximately 1000 nucleotides underwent modification, yet the amino acid sequence remained unaltered. A Kozak sequence was incorporated after the upstream restriction site and *Nde* I and *BamH* I sites were introduced. Highly conserved epitopes consisting of the A α-helix of HA, the ectodomain of M2 and the HCA-2 of NA were connected into a continuous gene, and *Not* I and *Xho* I restriction sites were inserted 5' and 3' of the optimized gene (G3) sequence. The G3 epitopes (**Supplementary Table S2**) were chosen based on IEDB conservation scores and HLA-binding predictions (HLA-DR4/DR7, common in humans). The IBV HA gene and epitope gene G3, which had incorporated homology arms, were ligated into the pVAX1-IRES vector, using seamless cloning method. The cytomegalovirus (CMV) promoter and internal ribosome entry site (IRES) sequence separately initiated two segments of the inserted gene.

### 2.3 *In vitro* transfection

Plasmid DNA was transfected into HEK293 or BMDC cells using the Advanced DNA/RNA Transfection Reagent (ZETA Life, USA). Briefly, 3×10^5^ cells were inoculated into 6-well plates. Following an overnight incubation, aliquots of a solution containing 2.5 μg of plasmid DNA and Advanced DNA/RNA Transfection Reagent were added to each well. The pVAX-1 vector was used as negative control. Following a 36-h incubation at 37°C, the cells were collected and transfected using Cell Lysis Buffer (Beyotime Biotechnology, China). The supernatant of the lysates was collected for western blot analysis as described next. Materials examined by an immunofluorescence assay as described below were washed and fixed with 4% paraformaldehyde in a 6-well plate.

### 2.4 Western blot

The cell lysates were collected and the proteins resolved by 10% SDS-PAGE. The proteins were subsequently transferred to nitrocellulose membranes for immunoblotting. The membranes were blocked using 3% bovine serum albumin (BSA) for 1 h. Rabbit anti-IBV HA protein antibody (GeneTex, USA), rabbit anti-IAV M2 protein antibody (GeneTex, USA), and rabbit anti β-actin antibody (Cell Signaling Technology, USA) were each used at a 1:1000 dilution. Horseradish peroxidase (HRP)-labelled goat anti-rabbit IgG antibody (Beyotime Biotechnology, China) was used at a 1:25000 dilution. PBS as the MOCK control. The bands were visualized using SuperSignal West Femto (Thermo Fisher Scientific, USA).

### 2.5 Immunofluorescence assay

The cells were permeabilized with 0.5% Triton X-100 after fixation, then blocked in 3% BSA for 2 h. The primary antibody used in this experiment was the same antibody used in western blotting, while fluorescein isothiocyanate (FITC)-labelled goat anti-rabbit IgG antibody (Beyotime Biotechnology, China) was used at a 1:1,000 dilution as the secondary antibody. Nuclei were stained with 4',6-diamidino-2-phenylindole (DAPI; Sigma-Aldrich, Germany), and images were obtained using an EVOS M5000 microscope (Invitrogen, USA).

### 2.6 Immunization

**Figure 1:**
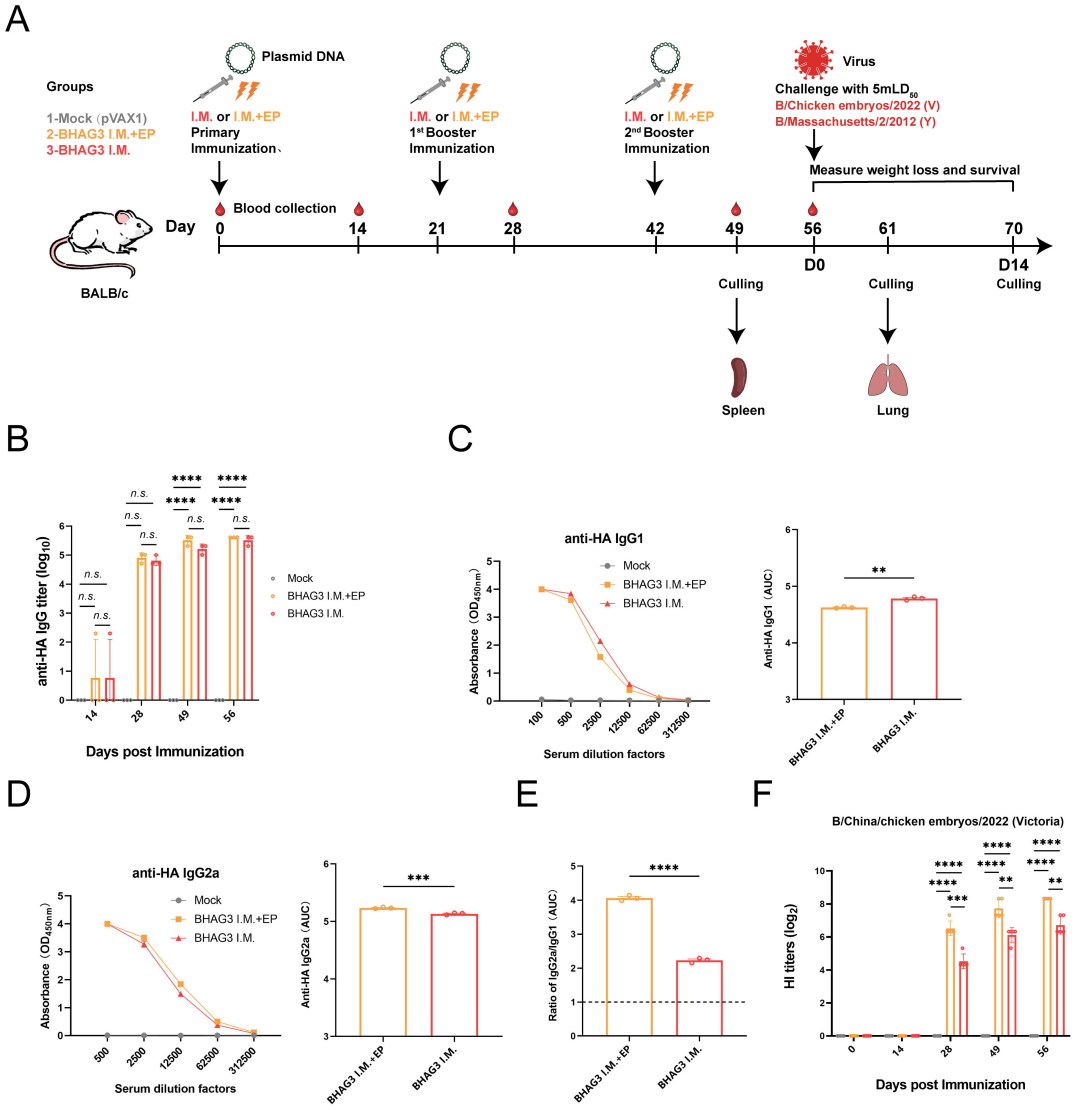
Schematic illustration of the immunization strategy in mice and humoral immunity induced by BHAG3. The mice (n=19) in the BHAG3 I.M. group were injected intramuscularly with 100 μg of vaccine, while the mice in the BHAG3 I.M. + EP group additionally received electroporation (EP) *via* electric pulses immediately after intramuscular injection 100 μg of vaccine **(A)**. The animals in the Mock group were injected with 100 μg of pVAX1 followed by EP. On day 14 following the final immunization, mice were infected intranasally with IBV (mLD_50_ = 5). Sera were collected 0, 14, 28, 49, and 56 days after the initial immunization. The sera from the immunized mice (n=3) were analyzed for specific IgG potency **(B)** by ELISA, and the IgG1 endpoint potency **(C)** and IgG2a endpoint potency **(D)** were assayed, with the IgG2c/IgG1 ratios subsequently calculated **(E)**. The presence of HI antibodies in the immune mice (n=5) was detected using the B/Victoria strain as the antigen **(F)**. Statistical analyses were performed using one-way ANOVA. Two-way ANOVA was used for comparison antibody titers among different groups within the different phases. (**P < 0.01, ***P < 0.001, ****P < 0.0001). n.s., not significant.

Female, 6-8-week-old Balb/c mice were immunized by intramuscular injection of BHAG3 (100 μg) and introduced by intramuscular injection followed by electroporation (BHAG3 I.M. + EP) or an equivalent amount of pVAX1 (Mock) *via* intramuscular injection. The EP was applied to the injection site immediately following injection by six 10-ms pulses using a two-electrode array at a depth of 5 mm. Electric pulses were delivered by the Advaccine Biopharmaceutics (Suzhou) Co. LTD. (China). Intramuscular injection of BHAG3 in the absence of EP (BHAG3 I.M.) was used as a matched control (**Figure 1A**).

The mice were vaccinated *via* a sterile insulin syringe (0.3×8 mm, U-40/30G) into both hind legs. The first and second booster was given 21 and 42 days, respectively, after the initial vaccination.

### 2.7 Enzyme-linked immunosorbent assay

To detect IBV HA-specific IgG and its subtypes, the ELISA was performed. The assay used 96-well polystyrene plates (Corning, USA) coated with IBV HA protein (Sino Biological, China) overnight at 4°C. After coating, the plates were washed three times with phosphate-buffered saline containing Tween (PBST). Five percent skim milk (BD, USA) was added to each well and incubated for 2 h at 37°C. After washing, plates were incubated with 2-fold serial dilutions of mouse sera starting from 1:100 and incubated at 37°C for 1 h. Following five washes in PBST, the plate was incubated for 45 min at 37°C with HRP-conjugated goat anti-mouse IgG (ZSGB, China), IgG1, or IgG2a antibody (Abcam, city, UK). Following five washes with PBST, 3,3,5,5′-tetramethylbenzidine (TMB; Sigma-Aldrich, Germany) was added to facilitate color development. The reaction was stopped by adding 2 mol/L H_2_SO_4_ at the appropriate time, and the optical density was measured at 450 nm using a 10M multimode microplate reader (Tecan, Switzerland).

### 2.8 Flow cytometry assay

Seven days after the final vaccination, the spleens of the mice were harvested. To harvest spleen, BALB/c mice were euthanized by cervical dislocation after being anesthetized with an intraperitoneal injection of tribromoethanol (2.01%, 20 μL/g body weight). A single-cell suspension was prepared and stimulated with inactivated influenza virus and treated with a solution of Brefeldin A for 6 h. Following stimulation, the cells were washed with PBS buffer and stained with CY5-coupled anti-mouse CD3, FITC-coupled anti-mouse CD4 and APC-coupled anti-mouse CD8a antibodies (BioLegend, USA). Following staining, cells were fixed using Cytofix/Cytoperm™ solution (BD, USA), divided into three portions and labelled using PE-coupled anti-mouse IFN-γ antibody (BioLegend, USA), PE-coupled anti-mouse IL-2 antibody (BioLegend, USA), or PE-coupled anti-mouse IL-4 antibody (BioLegend, USA), respectively. Subsequently, the stained cells were washed twice and analyzed using flow cytometry (Beckman Coulter, USA) to detect antigen-specific CD4^+^ and CD8^+^ T-lymphocyte-mediated immune responses.

### 2.9 Hemagglutination inhibition assay

The assay was performed as previously described (27, 28). Samples of sera were treated with receptor-destroying enzyme II (Denka Seiken, Japan) and diluted fivefold prior to the detection of HI in 96-well V-bottom microplates. The serum samples were adsorbed with 5% erythrocytes before testing. Each test sample was incubated for 30 min at room temperature with 4 HA units of virus (B/Chicken embryos/2022 or B/Massachusetts/2/2012), then mixed with 1% chicken red blood cells and further incubated for a further 30 min. The HI titer was defined as the reciprocal of the maximum serum dilution that inhibited viral hemagglutination.

### 2.10 Immunogenicity and protective efficacy in mice

Each group of BALB/c mice was divided into subgroups for challenge with either B/Chicken embryos/2022 (Victoria) (GenBank: OR775570) or B/Massachusetts/2/2012 (Yamagata) (GenBank: MT056027). The amino acid similarity between the Victoria lineage virus challenge variant used and the HA variant contained in the vaccine was 99.7%. Additionally, the amino acid similarity was 93% between the Yamagata lineage virus challenge variant used and the HA variant contained in the vaccine. The immunized mice were anesthetized with tribromoethanol and then challenged by *via* nasal installation of 50 µL of viral suspension, with an approximate dose of 5 mouse lethal dose 50 (5 × mLD_50_ = 1 × 10^4.5^ TCID_50_). Body weight and mortality rates were monitored for 2 weeks. In another experiment, three mice were euthanized 5 days after challenge. Prior to euthanasia, mice were anesthetized *via* intraperitoneal injection with tribromoethanol (2.01%, 20 μL/g body weight) and then euthanized by cervical dislocation. The lung tissues were harvested for virus titration and histopathological analysis. In accordance with the ethical guidelines for animal experimentation, a humane endpoint of 25% weight loss was used in this study (21).

### 2.11 Detection of viral RNA load and inflammatory factors in lung tissue

Five days following the viral challenge, the lungs of the mice were collected to measure the viral load. Viral RNA was extracted from the supernatant of the homogenate using the RaPure Viral RNA/DNA Kit (Magen, China) as described by the manufacturer. Viral RNA in lung tissue was quantified using the HiScript II U^+^ One Step qRT-PCR Probe Kit (Vazyme, China). The sequences of the primers and probe-specific used are provided as supplementary data (**Supplementary Table S1**).

To determine whether the vaccine could attenuate the inflammatory response, Total protein was extracted from the homogenous lung tissue using the ProteinExt® Mammalian Total Protein Extraction Kit (TransGen, China) exactly as described by the manufacturer. The expressions of pro-inflammatory cytokines, including IL-1β, IL-6, IFN-γ, and TNF-α, were assessed by ELISA kits (R&D, USA) in lung tissue from mice infected with B/Victoria and B/Yamagata viruses.

### 2.12 Histological analysis and immunohistochemistry

Lungs were fixed in 10% formalin for approximately 12 h and subsequently embedded in paraffin blocks. The lung sections were stained with hematoxylin and eosin (H&E) prior to being scored for the presence of pulmonary hemorrhage, inflammatory cell infiltration, hyaline membrane formation, alveolar atrophy and collapse, alveolar wall thickening, pulmonary edema, and hemorrhage on a scale from 0–3. Immunohistochemistry was performed on the sections using rabbit anti-IBV NP antibody (GeneTex, USA). The percentage of positive cells was quantified.

### 2.13 *In vitro* assay for activation and uptake capacity of dendritic cells

The relationship between BHAG3-induced antibody production and early activation of the innate immune system was assessed. Bone marrow of 6-week-old mice was extracted and BMDCs isolated. DCs were stimulated with PBS, lipopolysaccharide (LPS), or BHAG3 at 37°C. The control was 4°C conditioned treatment. FITC-dextran treatment was added at the end of the stimulation. The percentage of DCs that phagocytosed FITC-dextran was determined by flow cytometry to indirectly assess the phagocytosis ability of DCs. The delivery capacity of DCs was evaluated using the mixed lymphocyte reaction. The lymphocyte stimulation index was calculated by adding CCK-8 after 36 h of co-culture and measuring the absorbance at 450 nm. The percentage of cells expressing CD40, CD80, CD86, and MHC-II surface markers (all from BioLegend, USA) was determined by flow cytometry following staining of antigen-stimulated DCs with antibodies against specific DC markers. The expression levels of inflammatory factors in culture supernatants of antigen-stimulated DCs were analyzed using ELISA kits (R&D, USA).

### 2.14 Statistical analysis

Statistical analyses were performed using the Prism 8.0.2 software (GraphPad, USA). One-way analysis of variance (ANOVA) was used for the comparison among multiple (>2) groups. Two-way ANOVA was used for comparison of antibody titers among different groups within the different phases. The antibody titers were compared by performing log-transformed values. *P* values <0.05, < 0.01, < 0.001, and < 0.0001 were considered statistically significant depending on the experiment.

## 3 Results

### 3.1 Construction of DNA plasmids and verification of protein expression

**Figure 2:**
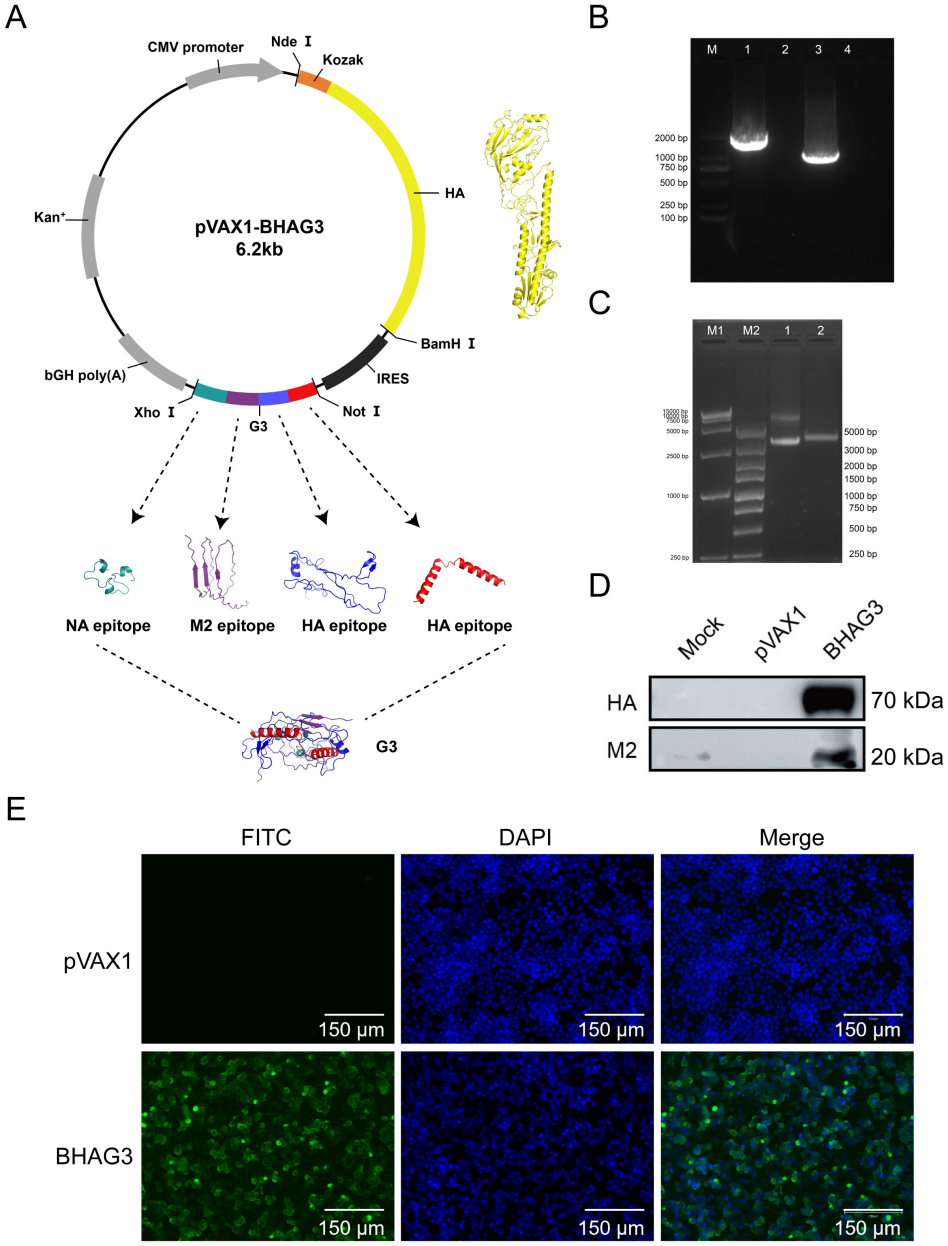
Construction and identification of DNA plasmid pVAX1-BHAG3. **(A)** Schematic design of the DNA vaccine construct. The predicated three-dimensional structure of natural HA. Tandem antigenic epitope (G3) comprised HA, M2, and NA peptide-encoding genes fused in tandem. The sequences were submitted to the AlphaFold 2.0 server to construct the protein structure. Visualization of the model was performed using PyMOL software. **(B)** PCR products of B/Victoria HA full-length (lane 1) and G3 antigenic epitope (lane 3) were analyzed using electropherograms. **(C)** BHAG3 (lane 1) and double enzyme digestion (*Nde* I and *Xho* I) products of BHAG3 (lane 2) were analyzed using electropherograms. **(D)** Following transfection of HEK293 cells with BHAG3, protein expression was confirmed by protein blotting using rabbit anti-HA protein antibody or rabbit anti-M2 protein antibody. **(E)** The expression of the protein was verified by indirect immunofluorescence. The transfected cells were stained with rabbit anti-HA protein antibody and FITC-labelled goat anti-rabbit IgG antibody (green), and the nuclei were re-stained with DAPI (blue). The scale bars are depicted in each image.

To develop a safe and efficient IBV vaccine, seamless cloning was used to construct a recombinant DNA vector that carried the full-length IBV HA gene and the G3 genes encoding the HA, M2e, and NA antigenic epitopes (**Figure 2A**). The description of the various epitopes selected was in **Supplementary Table S2**. The successful insertion of the genes was verified by PCR (**Figure 2B**) and double-enzymatic digestion analysis and sequencing (**Figure 2C**). The expression of the HA and M2 proteins in the constructs was detected using western blotting (**Figure 2D**). The expression of the HA protein was also detected using the immunofluorescence assay (IFA) (**Figure 2E**). The findings demonstrated the successful construction of a recombinant plasmid co-expressing the IBV HA gene and epitope gene G3 were successfully constructed in this study. The plasmid was designated BHAG3.

### 3.2 Immunization with BHAG3 induces antigen-specific humoral responses

The immune responses elicited by BHAG3 in mice were assessed. Serum samples obtained at different and specific times were analyzed to evaluate the potency of specific antibodies. The findings indicated a pronounced IBV HA IgG specific response (**Figure 1B**). Analysis of IgG subtypes revealed that BHAG3 triggered a predominantly IgG2a immune response, whereas the IgG2a potency induced by BHAG3 I.M. + EP was 1.262 times higher than that induced by BHAG3 I.M. (**Figures 1C–E**). Moreover, the IgG2a/IgG1 ratio in both vaccine modalities exceeded 1.0, indicating that BHAG3 significantly increased IgG2a levels.

To further evaluate the antibody response to the vaccine, we examined HI antibodies in the collected sera. After the second immunization, the levels of vaccine-induced HI antibodies were significantly elevated, and EP further enhanced this antibody response. The peak geometric mean titer (GMT) induced by BHAG3 I.M. reached 1:105.6, while the GMT for BHAG3 I.M. + EP reached 1:320 at 56 days following the initial immunization (**Figure 1F**). Additionally, HI antibodies in the sera from the BHAG3-immunized group exhibited strain-specificity, with inhibition observed only against strains with matching HA gene profiles, but not against strains with mismatched HA gene profiles (**Supplementary Table S3**).

### 3.3 Immunization with BHAG3 induces cellular immune responses

**Figure 3:**
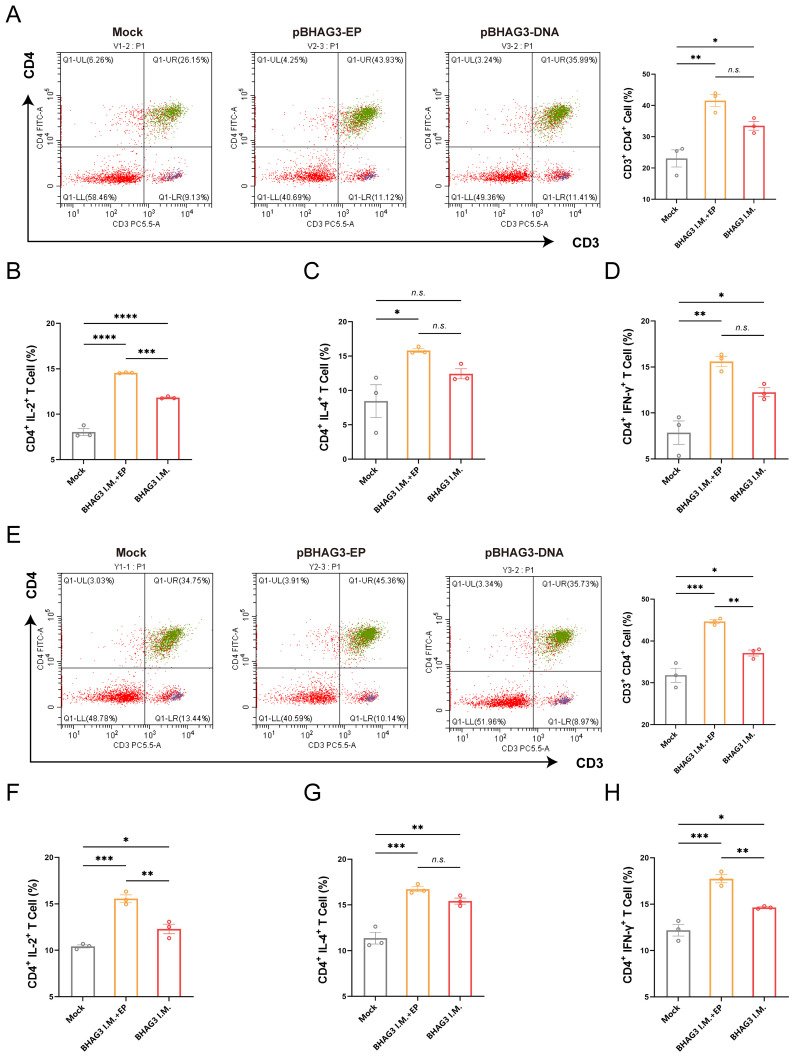
Immunization with BHAG3 induces cellular immune responses. Splenocytes collected from mice (n=3) 7 days following the final vaccination were stimulated with B/Victoria **(A-D)** or B/Yamagata **(E-H)** inactivated virus. The cells were assessed by flow cytometry for CD4^+^ T-cell proliferation **(A, E)** and the production of cytokines IL-2 **(B, F)**, IL-4 **(C, G)** and IFN-γ **(D, H)**. Statistical analyses were performed using one-way ANOVA. (*P < 0.05, **P < 0.01, ***P < 0.001, ****P < 0.0001). n.s., not significant.

To investigate the vaccine-induced cellular immune response, spleen lymphocytes were isolated from mice 7 days following the final vaccination. The proportion of activated T cells and cytokine expression were determined by flow cytometry. They demonstrated BHAG3-induced antigen-specific CD4^+^ T-cell responses (**Figure 3A**). No significant enhancement was observed in CD8^+^ T-cell responses. Furthermore, the cytokine results demonstrated that BHAG3 induced a notable elevation in the expressions of IL-2, IL-4, and IFN-γ expression (**Figures 3B–D**). In particular, BHAG3 I.M. + EP produced a 0.23-fold increase in IL-2 levels relative to BHAG3 I.M. These observations impllid that BHAG3 I.M. + EP may potentially elicit more robust cellular immune responses. Notably, the strategy also triggered cross-reactive T-cell immunity in response to stimuli from the B/Yamagata lineage of IBV, implying potential cross-protection against heterologous influenza attacks (**Figures 3E–H**). The data demonstrated that immunization with BHAG3 I.M. + EP elicited robust CD4^+^ T cell responses in BALB/c mice.

### 3.4 Evaluation of the protective effect against different lineages of IBV

**Figure 4:**
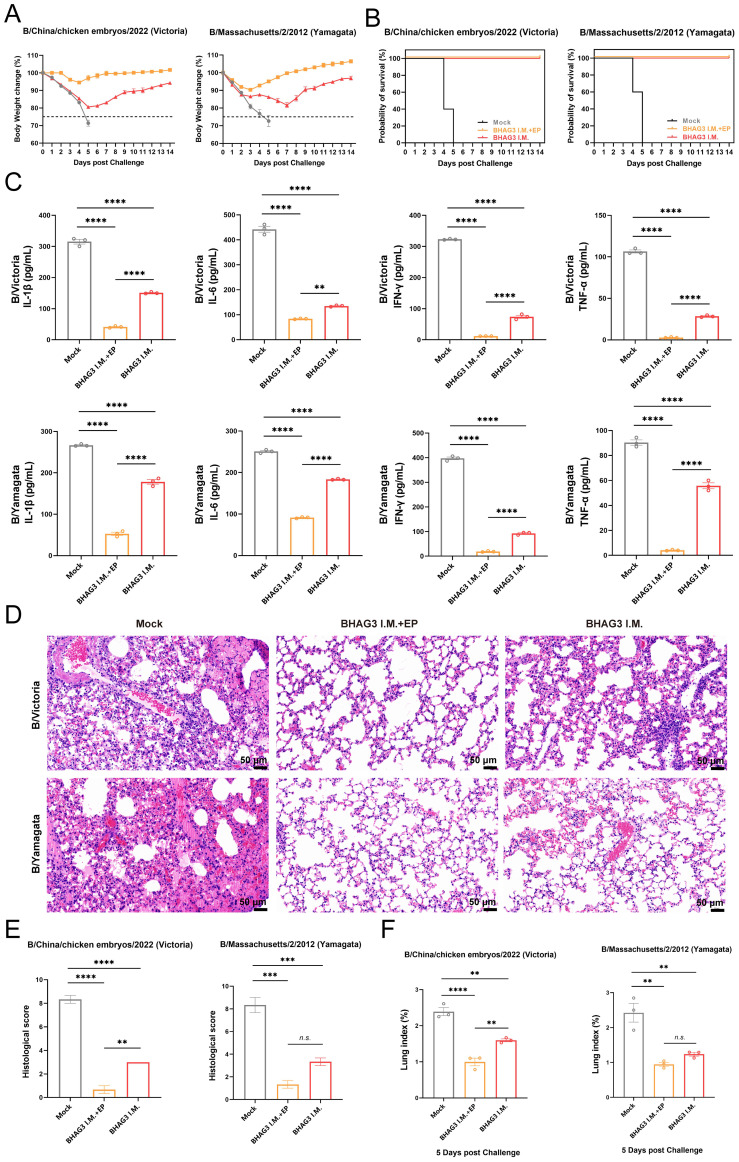
Evaluation of the protective effect against different lineages of influenza B virus. Mice were challenged I.N. with B/Chicken embryos/2022 (Victoria) strain or B/Massachusetts/2/2012 (Yamagata) strain at 5 mLD_50_ and monitored for 2 weeks for body weight loss **(A)** and survival **(B)** (n=5). In another experiment, lung tissues were collected from mice on 5th day post-challenge (n=3). A portion of lung tissue was used to extract total proteins, and changes in inflammatory factors IL-1, IL-6, IFN-γ and TNF-α were analyzed by ELISA **(C)**. A portion of lung tissue was embedded in paraffin (n=3), then the lung tissue was fixed, and pathological sections were made for observation by H&E staining **(D)**, and the H&E results were scored clinically **(E)**. Prior to the experiment, the total weight of the lung tissue (n=3) was recorded to calculate the lung index **(F)**. Statistical analyses were performed using one-way ANOVA. (**P < 0.01, ***P < 0.001, ****P < 0.0001). n.s., not significant.

Although the B/Yamagata lineage has not been widely detected in recent years, it serves as a relevant model for assessing cross-lineage immunity. To evaluate the protective efficacy of BHAG3, the incidence of morbidity and mortality from weight loss was monitored over a 14-day period. All mice in the Mock group that were infected by the B/Victoria and B/Yamagata IBV lineages died of the viral infection within 5 days. The mice in the BHAG3 I.M. + EP group exhibited slight weight loss (up to 8% loss) on days 3−4 following infection by B/Victoria, which was followed by growth and weight gain. Mice infected by B/Yamagata also experienced transient weight loss (up to 9% loss) on days 1−3 days after infection, followed by growth and weight gain. All mice in the BHAG3 I.M. + EP group survived the infection by both B/Victoria and B/Yamagata IBV. Mice in the BHAG3 I.M. group exhibited significant and sustained weight loss (up to 20% loss) following infection by B/Victoria and B/Yamagata IBV (**Figure 4A**). However, these mice were protected with 100% survival (**Figure 4B**). This demonstrated that the vaccine could provide cross-protection against a heterologous lineage, even in the absence of high-titer HI antibodies against the challenge strain, underscoring the potential of the conserved epitope-based approach to broaden protection.

The expression of inflammatory cytokines exacerbates symptoms and inflammatory responses that develop upon infection with influenza virus. In the present study, mouse lung tissues of comparable dimensions were pulverized, and proteins were extracted to detect inflammatory cytokines. The four inflammation-associated factors IL-1β, IL-6, IFN-γ, and tumor necrosis factor-alpha (TNF-α), exhibited a similar trend (**Figure 4C**) and their levels were significantly lower in both BHAG3-immunized groups compared to the Mock group (*P* < 0.0001). Notably, the BHAG3 I.M. + EP group exhibited a lower expression of inflammatory factors compared to the BHAG3 I.M. group. Additionally, histological analysis revealed that mice in the Mock group exhibited significant lung tissue damage following infection with both B/Victoria and B/Yamagata IBV. This damage manifested as edema, hemorrhage, thickening of the alveolar wall, infiltration of inflammatory cells, and atrophy and collapse of the alveoli. In contrast, mice in the BHAG3 I.M. + EP group exhibited minimal abnormalities and a more defined alveolar wall structure. Lung structural integrity was maintained in mice in the BHAG3 I.M. group, although they displayed slight inflammatory cell infiltration and dilated and congested capillaries in the alveolar walls (**Figures 4D, E**). The lung tissues of the mice were weighed at the time of collection. The calculated lung index results were homologous with the histological scoring values (**Figure 4F**).

**Figure 5:**
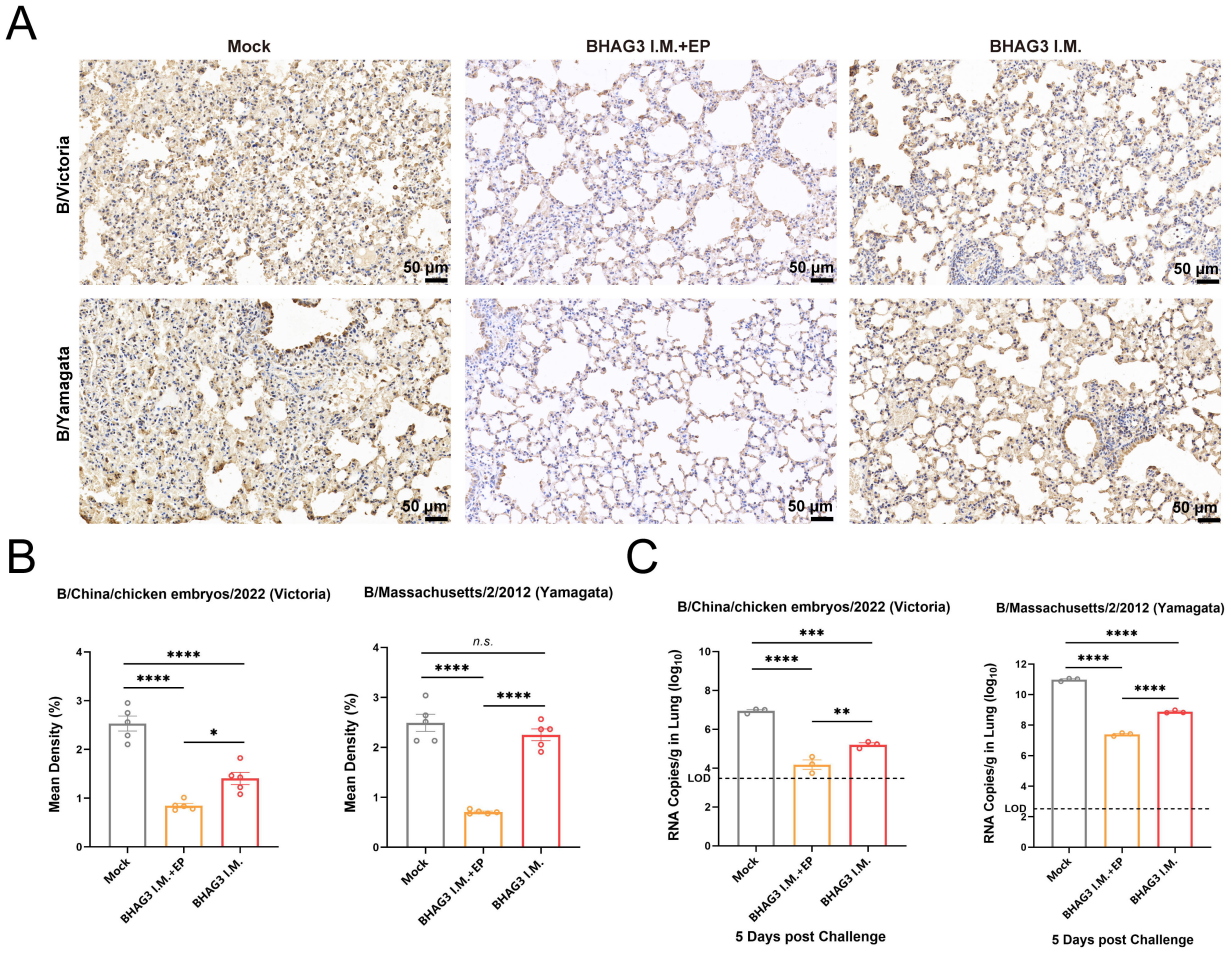
Vaccination reduces viral load in the lung. Lung tissues collected 5 days following virus challenge were fixed and pathological sections were made after paraffin embedding (n=3). Antigens in lung tissues were detected by immunohistochemistry **(A)** and the percentage of positive cells **(B)** in each group was compared. **(C)**Detection of B/Victoria or B/Yamagata viral load in lung tissue (n=3). The dotted line indicates the limit of detection. Statistical analyses were performed using one-way ANOVA. (*P < 0.05, **P < 0.01, ***P < 0.001, ****P < 0.0001). n.s., not significant.

Immunohistochemical analysis of mouse lung tissues revealed that immunization by both BHAG3 I.M. + EP and BHAG3 I.M. resulted in a notable reduction in the deposition of IBV NP. Moreover, the percentage of NP-positive cells in the BHAG3 I.M. + EP group following immunization was < 1%, indicating a greater ability to inhibit IBV replication (**Figures 5A, B**). To further confirm the ability of vaccination to clear IBV, the viral load in lung tissues was assessed by RT-qPCR. Viral loads of B/Victoria and B/Yamagata in lung tissues were significantly reduced in the BHAG3 I.M. group. The BHAG3 I.M. + EP group exhibited a nearly 1000-fold reduction in viral RNA load in lung tissue compared to the Mock group (**Figure 5C**; *P* < 0.0001). The results indicated that BHAG3 I.M. + EP provided comprehensive protection against both lethal doses of B/Victoria and B/Yamagata, while also effectively reducing the viral load in lung tissues. BHAG3 I.M. only provided partial protection against lethal doses of B/Victoria and B/Yamagata.

### 3.5 *In vitro* assay for activation and uptake capacity of DCs

**Figure 6:**
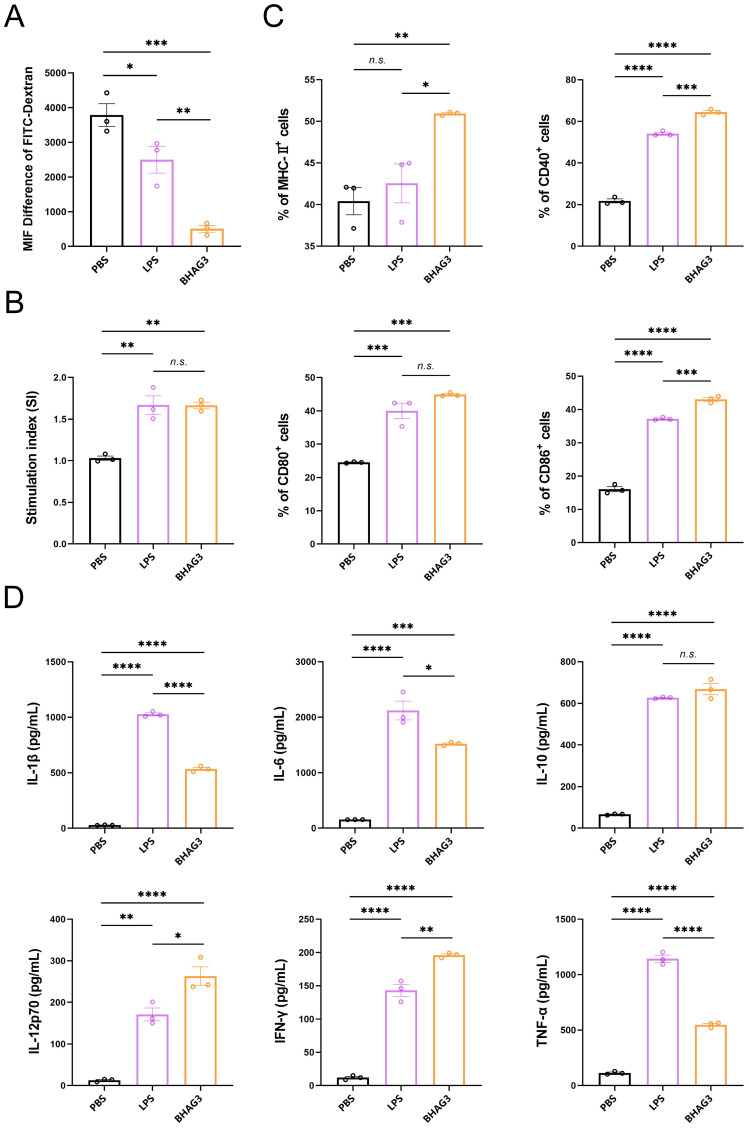
*In vitro* activation and uptake capacity of DCs. DCs were stimulated with PBS, LPS and BHAG3 at 37°C. The control was conditioned medium at 4°C. FITC-dextran treatment was added at the end of the stimulation. The percentage of DCs that phagocytosed FITC-dextran was analyzed by flow cytometry to indirectly assess the phagocytosis ability of DCs **(A)**. The delivery capacity of DCs was evaluated using antigen-stimulated DCs co-cultured with spleen lymphocytes **(B)**. The percentage of cell surface markers CD40, CD80, CD86 and MHC-II expression was detected by flow cytometry following staining antigen-stimulated DCs with antibodies against specific DC markers **(C)**. The expression levels of inflammatory factors in culture supernatants of antigen-stimulated DCs were analyzed using ELISA **(D)**. Statistical analyses were performed using one-way ANOVA. (*P < 0.05, **P < 0.01, ***P < 0.001, ****P < 0.0001). n.s., not significant.

The objective of the *in vitro* experiments was to investigate the uptake and delivery capacity of BHAG3 by DCs and to analyze the co-stimulatory factors that are upregulated on the surface of DCs and the pro-inflammatory cytokines that are secreted by DCs following the uptake of BHAG3. The results demonstrated the uptake of BHAG3 and delivered by DCs in substantial quantities (**Figures 6A, B**). In comparison to the PBS group, BHAG3 significantly stimulated the expression of DC surface co-stimulatory molecules CD80 and CD86, and T-cell co-stimulatory molecules CD40 and major histocompatibility complex II (MHC-II) (**Figure 6C**). BHAG3 significantly increased the secretion of the TNF-α, IFN-γ, IL-1β, IL-6, IL-10, and IL-12p70 cytokines (**Figure 6D**). These findings indicated that BHAG3 activated the maturation of DCs and induced the release of a substantial number of pro-inflammatory cytokines, which in turn promoted the proliferation and differentiation of other immune cells and had the capacity to rapidly activate the innate immune response.

## 4 Discussion

In contrast to the diversity of IAV, IBV have had limited evolution and are divided into the Victoria and Yamagata lineages. Despite the development of numerous influenza vaccine strategies (29), a universal influenza B vaccine remains unavailable for clinical use, with the exception of inactivated and subunit vaccines. The selection of appropriate immunogens is of paramount importance in the process of vaccine design. The authorized vaccines against influenza elicit the production of antibodies directed against the HA surface protein of the influenza virus. However, HA mutations can result in immune evasion, which diminishes vaccine efficacy (30–32). In this study, we selected a DNA vaccine platform that enabled the expeditious replacement of HA genes to align with prevalent strains. No alteration to the production technology was necessary following replacement (33). In recent years, vaccines based on novel vaccine platforms have begun to target conserved antigens, including T-cell responses induced by conserved epitopes of the HA, NP, M1, and M2 proteins. Studies on IAV NP and M2 proteins have demonstrated that NP vaccination elicits protection through cellular immunity and that conserved epitopes in M2 can induce alloimmunity (34–40). Vaccines based on the NA protein have demonstrated considerable potential for providing comprehensive protection against IBV in animal models (18, 20, 41). Furthermore, some studies have sought to provide broad cross-protection against multiple IBV strains in mice by chimerizing HA or utilizing the HA0 peptide on HA as an immunogen (22, 42–47).

In this study, conserved epitopes were incorporated as immunogens. Given the inherent challenges associated with the expression of multiple genes in tandem, we sought to identify a solution that would facilitate the separate expression of the HA gene and the epitope. To this end, we employed the pVAX1-IRES construct to enable the serial expression of the epitopes (**Figure 2A**). This approach was deemed the most suitable way of addressing the limitations of multiple gene expression in tandem (48–52). A broadly protective IBV vaccine against multiple antigens that was developed (21), included HA, NA, and NP of B/Victoria, M2, and a pentavalent mRNA-LNP vaccine against HA of B/Yamagata. The vaccine induced a broad protective immune response and proved to be more effective than the monovalent constructs, providing a foundation for further research into vaccination protocols against this combination of antigens.

The inclusion of conserved epitopes from NA and M2e in the G3 construct is a key feature of our vaccine design, likely contributing to the observed cross-protection. While HA-specific antibodies are crucial for neutralization, they are often strain-specific. In contrast, immunity against the more conserved NA protein has been shown to reduce viral replication and shedding, and can provide broad cross-protection (18, 20). Similarly, the extracellular domain of the M2 protein (M2e) can elicit non-neutralizing antibodies that mediate effector functions such as antibody-dependent cellular cytotoxicity (ADCC) and macrophage-mediated phagocytosis (16, 39). The robust Th1-skewed immune response induced by our vaccine, characterized by high IgG2a titers, is particularly effective at engaging these Fc receptor-dependent mechanisms. Therefore, the coordinated responses against HA, NA, and M2e likely acted synergistically to achieve the comprehensive protection and reduction in viral load observed against the heterologous B/Yamagata challenge.

The findings of our study indicated that BHAG3 elicited cross-reactive immune responses against both B/Victoria and B/Yamagata. Following stimulation of spleen lymphocytes with each of the two inactivated viruses, the percentage of CD4^+^ T cells in the BHAG3-immunized group was significantly increased in comparison with the Mock group (**Figures 3A, E**). However, no significant difference was observed in the percentage of CD8^+^ T cells between all groups. The recombinant DNA and vesicular stomatitis virus vectored vaccines constructed in a prior study (53) elicited a robust NA1-specific CD4^+^ T and CD8^+^ T-cell response. This was due to the inclusion of CD4 and CD8 T cell epitopes in the chimeric antigens, which induced a pronounced immune response. In a separate study, Kim et al. demonstrated that CD4^+^ T cells play a more significant role than CD8^+^ T cells in conferring cross-protection (54). This finding is consistent with the present results of the subsequent protection against challenge experiments (**Figure 4B**).

In mice, IgG1 and IgG2a are the most prevalent competitive antibody subtypes, representing Th2- and Th1-type immune responses, respectively. The ideal situation would likely be manufacturing a properly balanced Th2 and Th1 reaction, adjusted to the immune challenges (55). The findings of our study demonstrate that the BHAG3 I.M. + EP mice displayed elevated levels of B/Victoria HA IgG antibodies and hemagglutination inhibition (HI) titers against B/Victoria, indicating that electroporation enhances the immunogenicity of the vaccinated vaccine (**Figures 1B, F**). However, the high immunization dose increased IgG antibody levels in all immunized groups, resulting in a nonsignificant difference between the I.M.+EP and I.M. Furthermore, BHAG3 I.M. + EP markedly elevated antibody titers against IgG1 and IgG2a, with the IgG2a/IgG1 ratio approaching 4.0 (**Figure 1E**). Immune responses with a Th1 bias mainly produce IgG2a antibodies. The efficacy of IgG2a antibodies in clearing virus infections is superior to that observed with IgG1 (56). Some studies have indicated that Th1-biased immune responses may offer a means of avoiding the risk of vaccine-associated enhanced respiratory disease, in comparison to Th2-biased immune responses (57, 58). We also noted a recent study by Zhang et al., where it was demonstrated that with a slight Th1-skew provided partial cross-protection, are not sufficient to completely protect against challenge (59). And in a study by Han et al., mNA1-based constructions induced a robust NA-specific Th1-dominated immune response, capable of eliciting a robust T-cell response and providing superior protection against homologous and heterologous influenza infection (53). It is consistent with the rationale for our strategy, G3 included HA, NA, M2 epitope which from H1, H3, H5, B/Victoria and B/Yamagata. It is designed to maximize coverage of T cell epitopes and thus induction of cross-reactive T cell responses.

Vaccination of mice with BHAG3 significantly increased survival (**Figure 4B**) after B/Victoria or B/Yamagata infection and reduced viral load (**Figure 5C**) and lesions in the lungs (**Figure 4D**). However, an increased incidence of morbidity was observed in mice within the BHAG3 I.M. group that had been inoculated with a non-matching lineage of HA (B/Yamagata). Furthermore, a recent study described that antibodies of the non-matching profile were non-neutralizing and did not aid in the control of viral titers, although virus titers were somewhat reduced (21), as observed in the present study. This was attributed to the protection provided by antibodies of the non-matching profile through effector functions (60). IgG2a binds to Fc receptors with considerable affinity (61), which results in the stimulation of antibody-dependent cell-mediated cytotoxicity and macrophage-mediated opsonophagocytosis (62, 63). It is conceivable that these effects may have played a role in the effective clearance of lung viral loads, inducing augmented levels of antibodies capable of recognizing cell surface-expressed viral antigens from diverse strains.

In this study, the immunogenicity of EP and injection of naked DNA was compared in mice. EP enhanced the immune response to the DNA vaccine, as expected (**Figure 1E**). The effectiveness of EP delivery in activating the T-cell response has been demonstrated (64–68). *In vivo* EP has proven suitable for use in large animals, including humans (69). EP was used to deliver DNA, eliminating the requirement for additional adjuvants and reducing costs. This strategy also mitigates the risk of adjuvant-induced allergic reactions.

The findings of this study may substantiate the efficacy of DNA vaccine as an immunization strategy, offering cross-protection against the IBV. Accordingly, we investigated the mechanism of action of its antigen delivery. DCs represent the primary cells of the antigen-presenting cell population, and are pivotal in the interaction between innate and adaptive immunity following infection or immunization (70). Consequently, bone marrow-derived DCs were isolated *in vitro*. The recognition and uptake of DNA vaccine by DCs was confirmed by phagocytosis and mixed lymphocyte reaction assays (**Figures 6A, B**). To further elucidate the mechanism, we tested the capacity of the DNA vaccine to activate DCs, as only activated DCs are able to perform antigen delivery (71). The surface-associated factors of DCs were detected by flow cytometry, which revealed a significant elevation in the levels of the T-cell co-stimulatory molecule MHC-II, and DC surface co-stimulatory molecules CD80 and CD86, compared to the control group (**Figure 6C**). Furthermore, the cytokine profile of the culture supernatants was analyzed. The results demonstrated that BHAG3 effectively stimulated DCs to secrete cytokines (**Figure 6D**). These cytokines included IL-1β, IL-6, and TNF-α, which are inflammatory; IL-10, which is anti-inflammatory; and IL-12p70 and IFN-γ, which can promote the polarization of Th1 cells (72). Additionally, it has been demonstrated that DC-derived TNF-α plays a role in the development of CD4^+^ T cells that secrete IL-10 and Th1 cytokines (73, 74). IL-6 is a cytokine that facilitates the differentiation of T follicular helper cells (75). Based on these results, it is conceivable that DNA vaccines can activate DCs, which are recognized and taken up by them for antigen delivery. Nevertheless, this study initially examined the mechanism of vaccine action *in vitro* but lacked pertinent *in vivo* evidence. This was an acknowledged limitation of this study. Further studies will seek to refine this mechanism, improve translational applications.

In our former experiment, an immunized group with only the HA ectodomain was established, and a live attenuated vaccine group was included as a control. However, neither group demonstrated the capacity to produce cross-protection. Notably, the live attenuated vaccine group also failed to provide adequate protection, owing to the substantial discrepancy between the antigen and the viral challenge strain. The finding shows indirect support for G3 in correlation with cross-protection. However, the antigenic peptide protein is too small to synthesize, we will overcome for future experiments and elucidate the immunological effects of antigenic epitopes.

However, our study is associated with several limitations. While *in vivo* electroporation has been demonstrated to be a highly effective method for enhancing the immunogenicity of the DNA vaccine in mice, its practical application for large-scale human vaccination presents challenges. These include the necessity for specialized delivery devices, potential user acceptability concerns, and increased procedural complexity compared to conventional intramuscular injection. Future research endeavors should prioritize the optimization of the delivery platform to enhance its translational potential. This could entail the exploration of innovative formulations, such as lipid nanoparticles, or alternative physical delivery methods that are more conducive to mass vaccination campaigns. Furthermore, the immunological mechanisms of the antigenic epitopes were primarily investigated *in vitro*; more *in vivo* evidence would strengthen our understanding of their contribution.

In conclusion, the development of a DNA vaccine targeting IBV yielded promising results. Immunization *via* EP induced potent and broadly protective immunity in mice, providing 100% protection against lethal challenges of two IBV lineages. These findings suggest that BHAG3 had good immunogenicity and induced broad cross-reactive T-cell immunity. Furthermore, the strategy employed in the vaccine’s construction will help the development of a promising universal vaccine skeleton that could provide broad protection against existing and emerging influenza viruses.

## Data Availability

The original contributions presented in the study are included in the article/**Supplementary Material**. Further inquiries can be directed to the corresponding author/s.
