# Supplementary material for: A DNA vaccine based on hemagglutinin and conserved epitopes of influenza B virus provides cross-lineage protection in mice
Source: Front Immunol. 2025 Oct 29;16:1645744. doi: 10.3389/fimmu.2025.1645744 (PMC12605444; doi:10.3389/fimmu.2025.1645744)
Supplement: Supplementary file 1 [file Table1.docx]

**Table S1.** List of primers and probe-specific sequences used in this study.

| Primer | Sequence (5’-3’) | Modification |
| --- | --- | --- |
| Forward | ATTTTGCAAAYCTCAAAGGAACA |  |
| Reverse | TTGTTCTRTCGTGCATTATAGG |  |
| Victoria Probe | TGGGYAGACCAAAATGCACRG | 5`VIC, 3`BHQ1 |
| Yamagata Probe | GCAGGCCAATGTGTGTGGGRA | 5`FAM, 3`BHQ1 |

**Table S2.** Source of epitope.

| Epitope | Source |
| --- | --- |
| HA epitope (Red) | H1/H3/B-Victoria |
| HA epitope (Blue) | B-Victoria/B-Yamagata |
| M2 epitope (Violet) | H1/H5/ B-Victoria |
| NA epitope (Green) | A/B |

**Table S3.** HI titers detected by B/Massachusetts/2/2012 (Yamagata).

| Days post immunization | HI titers |
| --- | --- |
| 0 | ＜1: 10 |
| 14 | ＜1: 10 |
| 28 | ＜1: 10 |
| 49 | ＜1: 10 |
| 56 | ＜1: 10 |

**Figure S1.** The cells were assessed by flow cytometry for CD8^+^ T-cell proliferation.


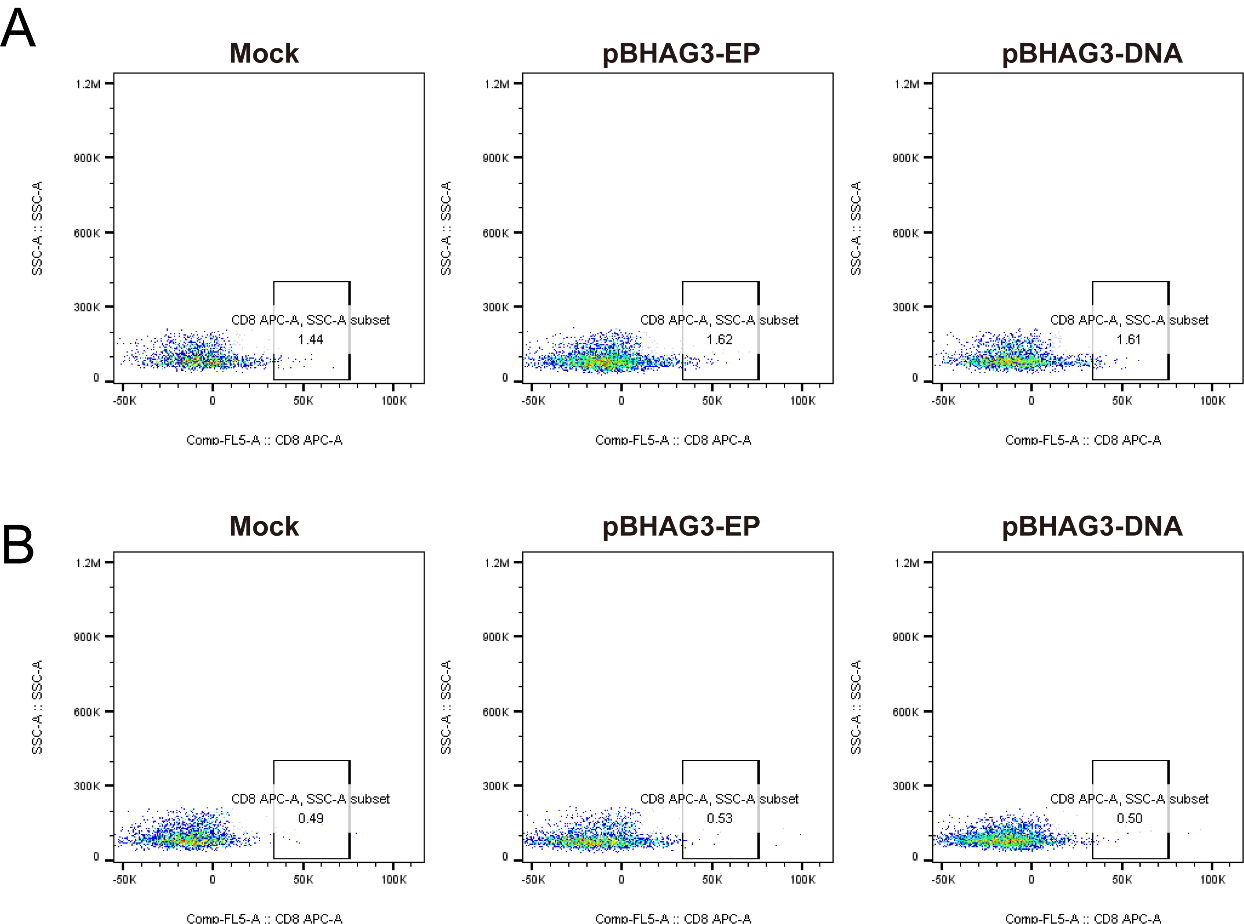


Splenocytes collected from mice (n=3) 7 days following the final vaccination were stimulated with B/Victoria (A) or B/Yamagata (B) inactivated virus.
